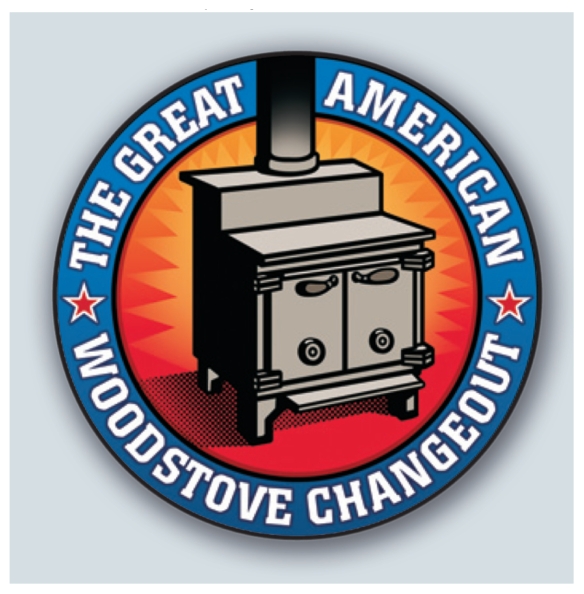# The Beat

**Published:** 2010-03

**Authors:** Erin E. Dooley

## EPA Releases Utilities’ Plans for Coal Ash Impoundment Safety

Since the December 2008 collapse of the Kingston Fossil Plant coal ash impoundment in Tennessee, which spilled 5.4 million yd^3^ of ash into the Emory River, the U.S. EPA has been conducting on-site assessments of impoundments at electric utilities across the country. On 4 February 2010 the agency released plans submitted by 22 utilities that detail how they are making their coal ash ponds safer through measures such as adding riprap and vegetation to strengthen earthen impoundment walls. In a press release the EPA said it is “continuing to review the reports and technical recommendations, and is working with the facilities to ensure that the recommendations are implemented in a timely manner.

**Figure:**
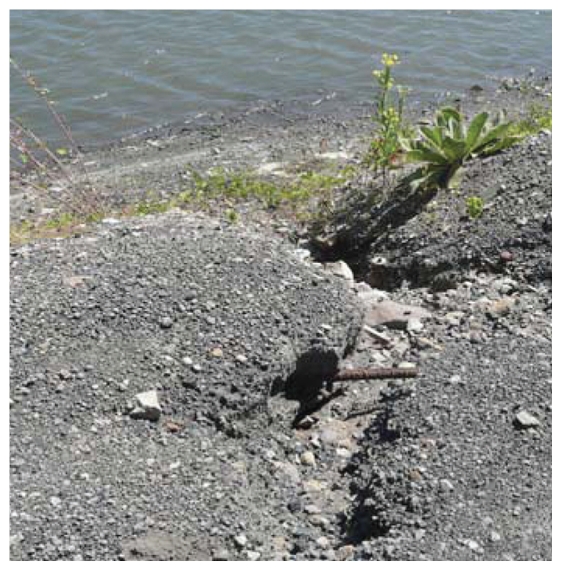
Erosion monitoring and control are key elements of several utilities’ plans.

## Rating the Sustainability of Roads

In January 2010 the University of Washington and engineering firm CH2M Hill released Greenroads™, a new system that rates the sustainability of road design and construction projects similarly to how programs such as LEED^®^ rate and certify buildings. Minimum requirements for Greenroads certification include a noise mitigation strategy and a life cycle energy and emissions analysis for paving materials. Extra points are awarded for voluntary measures such as avoiding light pollution, using permeable pavements to reduce stormwater runoff, and adding lanes for bicycles and pedestrians.

## New Lighting from Nanofibers

RTI International has developed a novel lighting technology that it says is 5 times more energy-efficient than incandescent bulbs and, unlike compact fluorescent lamps (CFLs), uses no mercury-containing components. RTI’s new technology, which was partially funded by the Department of Energy, pairs high-performance nanofiber-based reflectors with photoluminescent nanofibers to create light its developers say has better color rendering properties than is typically found with CFLs. The new technology could hit the market within 3–5 years.

## What Cd Means to CVD

A study by Junenette Peters and colleagues in the February 2010 issue of *Environmental Research* adds evidence to the idea that exposure to low levels of cadmium may be linked to cardiovascular disease. Using data from the National Health and Nutrition Examination Survey, the team found that a 50% increase in blood cadmium was associated with a 35% increase in stroke prevalence and a 48% increase in heart failure prevalence, while the same change in urinary cadmium was associated with 9% and 12% increases, respectively. Although smoking explained their findings in part, the researchers believe other exposures to cadmium, such as through food and metal and ceramic food containers, should be considered in both research and risk assessment for cardiovascular diseases.

**Figure:**
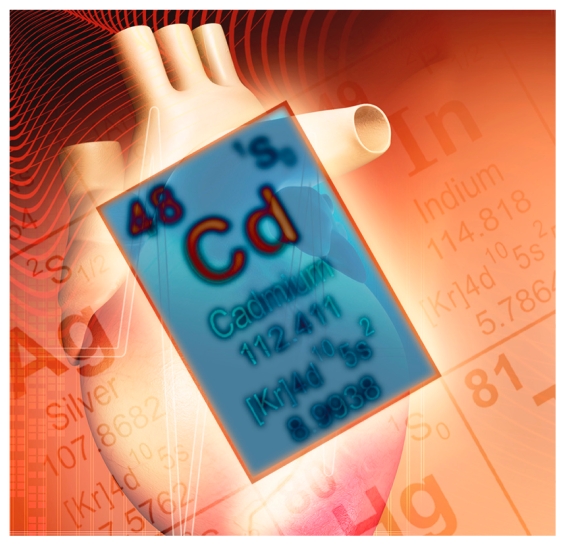
Low-level cadmium exposure may contribute to cardiovascular disease.

## China Completes First National Pollution Census

On 9 February 2010 the Chinese government announced it had completed the country’s first national census of pollution, a 2-year effort by almost 600,000 staff that mapped nearly 6 million pollution sources. This survey is the first time Chinese environmental authorities have been able to include agricultural sources of pollution in their data. Among other applications, these findings will help guide China’s next 5-year environmental protection plan to begin in 2011. However, the full survey results are available only to selected government officials. Environmental advocates are calling for the government to release details of the survey to the public.

## Communities Warm Up to Woodstove Changeout Programs

As part of the U.S. EPA’s Great American Woodstove Changeout campaign, more than 45 U.S. communities now provide financial incentives to residents who trade in old stoves for newer, less polluting models or pellet, gas, or propane appliances. Many of the older woodstoves in use can emit up to 10 times as much particulate matter as newer stoves, according to the EPA. As of January 2010, the agency estimated more than 13,000 woodstoves and fireplaces had been replaced under such programs, saving an estimated 248 tons of particulate matter emissions and $84 million in health benefits per year.

**Figure:**